# The Food Environment Perceptions Survey: development and piloting of a survey instrument in India and Cambodia to assess consumers’ interactions with diverse food environments

**DOI:** 10.1017/S1368980026102043

**Published:** 2026-02-13

**Authors:** Shauna Downs, Wiktoria Staromiejska, Serey Sok, Selena Ahmed, Elizabeth Fox, Anna Herforth, Suparna Ghosh-Jerath

**Affiliations:** 1 Department of Health Behavior, Society and Policy, Rutgers School of Public Health, Newark, NJ, USA; 2 Research Office, Royal University of Phnom Penh, Russian Federation Boulevard, Khan Toul Kork, Phnom Penh, Cambodia; 3 The Periodic Table of Food Initiative (PTFI), Sustainable Food Systems, American Heart Association, USA; 4 Department of Public & Ecosystem Health, Cornell University, Ithaca, NY, USA; 5 Division of Human Nutrition and Health, Wageningen University and Research, Wageningen, The Netherlands; 6 The George Institute for Global Health, Delhi, India

**Keywords:** Delphi method, Survey development, Food environment, Food systems

## Abstract

**Objective:**

The objective of this study was to develop, implement and refine a food environment survey to capture people’s perceptions of their food environments in low-and middle-income countries: the Food Environment Perceptions Survey (FEPS).

**Design:**

Identifying aspects of food environment to include drawing from existing survey instruments, a Delphi survey with food environment experts working globally, workshops with local experts in India and Cambodia, cognitive testing of the survey items and piloting the tools in diverse field settings in India and Cambodia.

**Setting:**

Rural, peri-urban and urban communities in India and Cambodia.

**Participants:**

Global food environment experts for the Delphi survey, food environment experts in India and Cambodia for workshops and a convenience sample of participants in India (n 44) and Cambodia (n 60) for FEPS piloting.

**Results:**

The FEPS underwent many iterations prior to piloting. The initial versions of the survey were long, leading us to remove questions and reconfigure the survey to streamline it. The workshop participants rated the revised survey versions relatively favourably. The final survey consists of 109 questions covering six sections: accessibility and availability (forty-eight questions); affordability (five questions), convenience (seventeen questions); quality and safety (three questions); information, promotion and labelling (sixteen questions); and an optional sustainability section (twenty questions). Based on pilot data, we found significant differences in how participants interacted with different food environment types across rural, peri-urban and urban transects.

**Conclusions:**

The finalised FEPS is a newly developed survey instrument that can be incorporated by other researchers to characterise diverse perceptions of food environments in low-and middle-income countries.

As consumers interface with the food system, food environments drive their decisions about what to eat, where to get it from and with whom to eat it^(1,2)^. Changes to food environments in low-and middle-income countries (LMIC) over the past few decades have been attributed to shifts in dietary intakes that have led to an increase in the prevalence of overweight and obesity and diet-related non-communicable diseases^(3–5)^. In some cases, changes in food environments have also coincided with reductions in the prevalence of undernutrition among children^(6,7)^, but not that of micronutrient deficiencies^(8,9)^.

Characterising the dimensions of diverse food environments in LMIC, including availability, affordability, convenience, quality and promotion and sustainability of foods^(2)^, can facilitate better design of interventions and policies that are tailored to the local context and people’s lived experiences. However, most of the tools that were designed to measure food environments were developed for high-income contexts^(2,10,11)^. In recent years, there has been a significant amount of progress in terms of developing assessments, tools and methodological approaches that are designed to capture objective measures of food environments in LMIC^(12–14)^, including in wild, cultivated and built spaces. This includes mapping vendors, collecting price data, observational checklists and other static measures about the food environment^(15)^.

Yet, it is also important to gain insight into how people navigate their personal food environments (i.e. ‘a set of individual-level dimensions’) and how they make food-related decisions within the contexts of their lived experiences^(16)^. This involves subjective measures since not everyone experiences a given food environment in the same way. Each individual has subjective interactions with food environments based on how much income or time a person has, their cultural identity, their physical capacity, whether they have a vehicle to access food sources and their personal preferences, among many others^(1,16)^. These lived experiences filter people’s engagement with objective measures in the food environment. Although a significant amount of qualitative research, including interviews, focus groups, photo elicitation techniques, etc., has been conducted to document how people’s lived experiences influence the way they navigate their food environments^(15–19)^, capturing this information through structured surveys could help provide more representative and comparable data of populations’ lived experiences complementing the findings of qualitative research in this space.

Existing surveys and questionnaires to capture the interface between the food environment and people’s lived experiences^(20,21)^ have been designed largely for high-income country contexts, without consideration of the multiple dimensions of diverse food environments in LMIC. Data that have measured food environments and people’s lived experiences in LMIC show that the way consumers interact with their food environments is markedly different than high-income settings^(10,15,16,22–25)^. Consumers in LMIC access different types of food environments; they produce or collect foods from natural sources, engage with vendors from informal settings, have different purchasing patterns due to a variety of reasons such as a lack of cold storage and emphasise food safety and vendor trustworthiness, among many other differences^(22)^. For these reasons, developing survey instruments that are tailored to the experiences of people living in LMIC is needed^(26,27)^.

The objective of this paper is to present the process of developing, implementing and refining a food environment survey to capture people’s perceptions of their food environments in LMIC: the Food Environment Perceptions Survey (FEPS). By describing the process of developing the FEPS, we provide transparency into the survey development and the steps taken to arrive at the finalised version.

## Methods

FEPS was co-created using an iterative process; Table 1 provides an overview of the steps taken as part of the survey development process. We (i) identified relevant aspects of food environments to include in the survey, (ii) drew from existing survey instruments and modified questions for suitability in diverse LMIC contexts, (iii) evaluated suitability and modified survey questions through a robust process of internal review and expert feedback and (iv) conducted cognitive and pilot testing in the field in diverse contexts in India and Cambodia and finalised the tool. The FEPS is one component of the Food Environment Toolbox that includes a suite of tools aimed at characterising food environments in LMIC settings.

**Table 1.** An overview of the steps taken as part of the Food Environment Perceptions Survey

Survey development step	Approach
Identification of food environment aspects to include in the survey	Identified aspects of the food environment to include in the survey based on existing food systems and food environment conceptual frameworks
Compilation of survey instrument items	Conducted targeted searches of the literature using Google Scholar and PubMed to identify existing survey instruments and studies that were considered relevant for the development of FEPS Key words included ‘food environment perceptions’ or ‘personal food environment’ and ‘survey’ or ‘questionnaire’ or ‘tool’ Contacted food environment experts who had developed or were in the process of developing surveys Compiled the specific survey items and the dimensions of the food environment that they addressed in an Excel file to be reviewed by the research team Made changes to the wording of questions to increase suitability to low-and middle-income country contexts
Delphi survey with food environment experts	Research team and Food Environment Toolbox Advisory Board identified food environment experts conducting research in LMIC Conducted two rounds of Delphi Survey using Qualtrics in December 2022 (round 1) and March 2023 (round 2) Experts rated the importance of including each survey item on a 5-point Likert scale ranging from ‘not a priority’ (1) to ‘essential’ (5) and were followed up with a question asking for feedback on suggested changes to the existing survey items Calculated median scores for survey instruments Questions scoring 4 or higher were retained in the subsequent survey round Questions scoring 3 or higher were reviewed alongside the qualitative feedback provided by experts and suggested modifications were applied to strengthen them, as relevant Qualitative feedback was compiled to formulate new questions to address gaps in the content that the questions covered, as highlighted by the Delphi experts
Cognitive testing	Conducted several rounds of cognitive testing in India and Cambodia Refined the survey based on feedback from the interviewee Participants were asked questions about their comprehension, retrieval/recall, their judgement and their response Research team discussed and incorporated feedback into the next version of the survey
Workshops with key food environment experts in India and Cambodia	Workshop participants were identified through discussions with our research team and their global and local networks Conducted workshops in India and Cambodia to ascertain feedback on the survey Workshop participants conducted a post-workshop survey to assess the survey’s strengths and weaknesses After the completion of each of the workshops, we made additional changes to the survey based on the feedback from the workshop participants
Pilot testing of FEPS	Programmed the survey using KoboToolbox to allow for electronic data collection and completed validation checks to ensure skip patterns were functioning as intended Conducted a pilot of the survey in rural, peri-urban and urban settings in India and Cambodia Met with the research team after every day of data collection to debrief and make changes to the survey to improve its implementation Examined data from the pilot to summarise logistical aspects of its implementation, we also reviewed the data to identify any questions that lacked variability in responses from participants or that would lead to challenges in data analysis and interpretation Finalised the survey based on piloting experience

FEPS, Food Environment Perceptions Survey; LMIC, low-and middle-income countries.

We obtained ethical approval from the Rutgers University Institutional Review Board and the institutional ethics committee of The George Institute for Global Health for this work. We also obtained ethical approval from the National Ethics Committee for Health Research in Cambodia and local authorities in Cambodia.

### Identification of aspects of food environments to include in the survey

The first step in our approach to developing the FEPS was to identify which aspects of how consumers interface with their food environments to include in the survey. We pulled from food environment and food system frameworks with a particular focus on LMIC to identify the constructs and food environment dimensions that interacted with individual factors^(2,16,28–30)^. More specifically, we used the following food environment dimensions to guide the survey development: accessibility (i.e. physical distance, time, space, place, etc.) and availability (i.e. the presence or absence of food sources and products)^(16)^, affordability, convenience, quality and promotion and sustainability of foods. The ways in which these dimensions influence food acquisition are influenced by individual factors, including economic (income and purchasing power), cognitive (information and knowledge), aspirational (desires, values, preferences) and situational (home and work environment, mobility, location, time) factors^(11,30)^. The survey content was designed to be flexible to capture consumers’ perceptions of their food environments and how they influence food acquisition within different types of food environments, including natural (i.e. wild and cultivated), built (i.e. formal and informal), supplemental food assistance and kin and community^(2,29)^.

### Compilation and initial modification of survey instrument items

We conducted a search and compiled existing survey instruments and studies that were considered relevant for the development of FEPS; relevant instruments and studies were determined as those that elicited people’s perceptions, experiences and knowledge of their food environments. We modified the wording and response options of existing items to be more relevant to diverse LMIC contexts and added new survey items when we perceived gaps in the information that was being collected for different food environment dimensions (e.g. accessibility and availability, affordability, convenience, quality and promotion and sustainability of foods). The adaptation of existing questions and the development of new ones were guided by key principles of survey development^(31,32)^.

### Delphi survey with food environment experts

The compiled survey items were then used in a Delphi survey with food environment experts. The Delphi method is a consensus-generating method of systematically gathering input from experts on a topic^(33)^; our goal was to reach consensus on survey items to include in the FEPS instrument. With the support of our Food Environment Toolbox Advisory Board, we identified experts with diverse backgrounds and experiences in evaluating food environments in LMIC. We sent the initial invitation of the Delphi survey to fifty-one food environment experts; a total of twenty-four experts participated in the first round of the survey. We sent the second-round survey to a total of twenty-nine experts (twenty-four from the first round plus five additional, noted below); a total of seventeen experts participated in the second round of the survey. We made a modification to the typical Delphi approach by inviting five additional experts who were unable to contribute to the first Delphi survey round due to time constraints to participate in the second round. While this is atypical of a standard Delphi method, we anticipated that the feedback that those experts would provide merited the modified approach and would still allow for the goals of the Delphi survey to be achieved (i.e. reaching consensus on survey items).

Descriptive statistics were used to calculate the median score for each of the items included in the survey^(34)^. We also used qualitative feedback to modify the wording of questions based on expert feedback. Survey questions that had sufficient support from experts and that maintained a consistent flow of the survey items were retained in the survey for cognitive testing of the draft survey tool. In addition to ascertaining feedback related to the different survey items, we also sought feedback on specific food groups to include in the FEPS as part of the Delphi survey. The food groups went through many iterations over the course of the project, which is described in additional detail elsewhere, given that we used the same food groups for the Toolbox overall^(35)^.

### Refining the tools based on cognitive testing and expert workshops

We conducted several rounds of cognitive testing of FEPS in India (in Hindi) and Cambodia (in Khmer). The first round of cognitive testing in India was conducted by members of the research team with people living in urban (*n* 5) and rural (*n* 5) areas between October and December 2023. After each round of cognitive testing, the feedback from the participants and the interviewer was summarised for each survey section. The rounds continued until no (or few) new insights were being revealed (i.e. saturation). The process was iterative, with each interview informing subsequent changes to the survey instrument, which were updated prior to the next round of cognitive testing.

In addition to feedback from participants of the cognitive testing interviews, we also elicited feedback from local food environment researchers and practitioners through a workshop in India (November 2023). Nineteen experts affiliated with universities (*n* 6), local and national research institutes (*n* 9), foundations (*n* 2) and UN agencies (*n* 2) attended the workshop in India. Members of our research team who conducted the cognitive testing presented their initial findings to workshop participants, which informed the discussion on changes that needed to be made to the survey. In addition to providing real-time feedback on the survey, workshop participants rated the FEPS in a post-workshop survey (rating range 1–3). Subsequent rounds of cognitive testing incorporated workshop participants’ feedback into a revised survey.

We conducted all rounds of cognitive testing and the workshop in India prior to conducting the testing in Cambodia; in Cambodia, our research team conducted cognitive testing with fifteen participants living in rural (*n* 5), peri-urban (*n* 3) and urban (*n* 7) settings between March and April 2024, following a similar process to what was described above for India cognitive testing interviews. We also conducted a workshop with key food environment researchers and practitioners in Cambodia (March 2024) to ascertain their feedback on the FEPS tool. In Cambodia, twenty-eight experts representing local universities (*n* 9), local and national research institutes (*n* 2), non-governmental organisations and civil society organisations (*n* 12), government agencies (*n* 3) and UN agencies (*n* 2) participated in the workshop. We shortened the workshop survey in Cambodia to reduce respondent burden and included a rating scale from 1–5 (strongly disagree to strongly agree), rather than the smaller scale (1–3) used in India, to capture more variation in workshop participants’ ratings. Any additional feedback was incorporated into the revised survey.

### Pilot testing of the Food Environment Perceptions Survey

After the cognitive testing and workshops with experts, we piloted the survey with a convenience sample of participants in rural (India *n* 21; Cambodia *n* 15), peri-urban (Narayanganj, a transitional area in Madhya Pradesh, India *n* 10; town on the outskirts of Phnom Penh, Cambodia *n* 15) and urban (Delhi, India *n* 13; Phnom Penh, Cambodia *n* 30) settings. The piloting took place between March and June 2024. We aimed to pilot the survey with fifteen people from each of the settings. This sample size is aligned with sample sizes for feasibility assessments^(36)^, given that the piloting was designed to assess the feasibility of implementing the survey instrument prior to implementing the survey with a larger sample size in future work and was not intended for hypothesis testing. In India, households in each community were approached by foot at random and asked if they would be willing to participate; in Cambodia, participants were identified through community focal points. In the rural and peri-urban settings, participants were generally low income; in the urban settings, we randomly selected participants regardless of income in Delhi and purposively selected low-and high-income participants in Phnom Penh.

### Analysis

We used descriptive statistics, using Microsoft Excel (16·87, 2024) to summarise results from the Delphi survey, the workshops and the pilot study. Using our pilot data and SPSS (Version 29), we conducted ANOVA for continuous variables and *χ* ^2^ tests (for categorical variables) to examine statistical differences in food environments accessed across rural, peri-urban and urban transects. A *P*-value of < 0·05 was considered statistically significant. In addition, we examined detailed notes from cognitive testing interviews and the workshop notes to thematically summarise the information related to the challenges of implementing the survey and experts’ perceptions of the survey, respectively.

## Results

### Compilation of survey instrument items

Online supplementary material, Supplemental Table 1 provides an overview of the manuscripts, reports and unpublished survey instruments that were reviewed to inform the development of the FEPS. Overall, we reviewed eighteen published manuscripts, some of which referred to several different survey instruments within them. In addition to these, we obtained three survey instruments^(26,27,37,38)^ from food environment researchers and practitioners working in LMIC that had not yet been published at the time we were compiling study instruments. We included 113 items from these surveys to be included in the Delphi survey.

### Delphi survey with food environment experts

**Figure 1.**
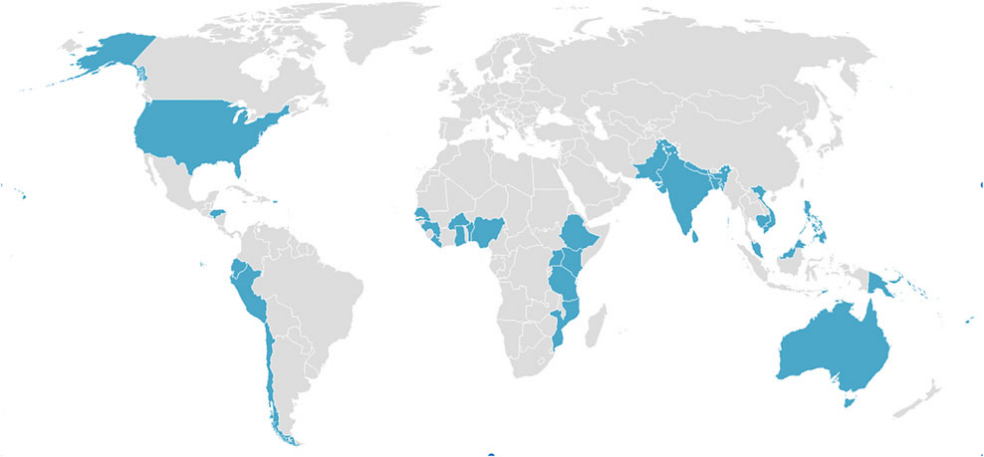
Countries in which Delphi participants conduct food environment work.

Overall, twenty-four of the invited participants (47 % response rate) completed the survey in round one. We sent the round two survey to twenty-nine participants, seventeen of whom completed the survey (59 % response rate). Delphi participants were mostly female (92 %), early or mid-career researchers (71 %), working in universities (58 %) and with nutrition as their primary discipline (63 %). Food environments and food systems were the primary specialties that participants used to describe themselves. All survey participants conducted food environment work in LMIC. Figure 1 depicts the specific countries (*n* 33) they reported working in.

Of the 113 survey items that were included in the first Delphi survey round, eighty-eight had a median score of 4 or above (78 %) and were included in the next round of the Delphi survey. Twenty-five survey items (22 %) had a median score below 4; all but one of these low-rated questions were removed in the survey’s next iteration. The survey item that had a score below 4 but was included in round 2 was modified based on its specific qualitative feedback from the Delphi participants and retained. We refined the remaining questions and added additional questions to address feedback from the Delphi participants about the gaps in the survey items that had been compiled.

In round 2, survey participants were asked to rate 102 survey items; this round determined which survey items would be included for cognitive testing. Survey items scoring 4 and 5 were automatically included (*n* 64, 63 %); one survey item (1 %) scored 2 and was automatically removed. Survey items scoring 3 (*n* 37; 36 %) were further reviewed for inclusion in the cognitive testing stage. Of the survey items that scored 3, 30 (81 %) were included in the subsequent version of the survey based on support from experts through qualitative feedback; the remaining questions (*n* 7) were removed from the survey after the second Delphi round due to their median score being three or below and their lack of support from experts. This resulted in a total of ninety-four survey items to be included in the survey for cognitive testing. In addition to the rating of survey items, we reviewed and incorporated qualitative feedback from round 2 for the retained questions prior to beginning cognitive testing of FEPS in India and Cambodia.

### Cognitive testing

Table 2 provides a summary of the responses to the cognitive testing questions for each of the survey sections in India. In the first round of the cognitive testing of FEPS in India, the first section of the survey that focused on food accessibility and availability took 90 min to complete. This led the team to reconfigure this section of the survey prior to conducting subsequent cognitive testing rounds. During the subsequent rounds of cognitive testing in India, the survey took an average of 74 min to complete with urban respondents and 135 min with rural respondents.

**Table 2.** Summary of cognitive testing findings of administering the Food Environment Perceptions Survey in India

Section 1. Accessibility and availability		
Cognitive stage	Urban	Rural
Comprehension	Respondents felt there were too many options; some declined to rephrase the question in their own words.	Declined to rephrase the question in their own words despite indicating they understood the question.
Recall	Easy to recall	A response wasn’t satisfactory due to recall bias
Judgement	Some respondents found the question difficult due to too many options.	Question was difficult for respondents.
Respond	Easy to respond	The respondent said that it was easy for them to answer the questions, but the enumerator did not agree.
Section 2. Affordability		
Cognitive stage	Urban	Rural
Comprehension	Easy to understand	Found difficulty understanding the questions; could not rephrase the question in their own words.
Recall	Easy to recall	Easy to recall
Judgement	Difficult to judge the cost of travel	Question was clear and quickly responded to.
Respond	Easy to respond	Easy to respond regarding fruits and vegetables; difficulty when responding to other food groups.
Section 3. Convenience		
Cognitive stage	Urban	Rural
Comprehension	Easy to understand	A respondent declined to rephrase the question in their own words despite indicating they understood the question.
Recall	Easy to recall	Easy to recall
Judgement	Question was clear and quickly responded to.	Question was clear and quickly responded to.
Respond	Easy to respond	Easy to respond

Additional changes to the survey were made based on feedback from workshop participants and input from the research team to further reduce the amount of time the survey took to complete. In the second round of cognitive testing, most sections of the survey required tweaks to wording and terms used, rather than complete overhauls of the survey sections, except for the food sovereignty section, which participants had difficulties responding to.

The cognitive testing in Cambodia was conducted several months after India. Between the India and Cambodia cognitive testing, we conducted workshops with experts in both countries and completed the rural piloting in India. For this reason, the survey instrument had undergone many changes, leading to the identification of fewer additional changes after the cognitive testing in Cambodia. In Cambodia, the survey took an average of 46 (rural setting) to 57 min (peri-urban setting) to complete. The amount of time taken to administer and complete the survey, particularly with participants from rural areas (i.e. the average time was reduced by 89 min between the countries). Overall, we identified small changes that improved survey delivery.

### Workshops with key food environment experts

**Figure 2.**
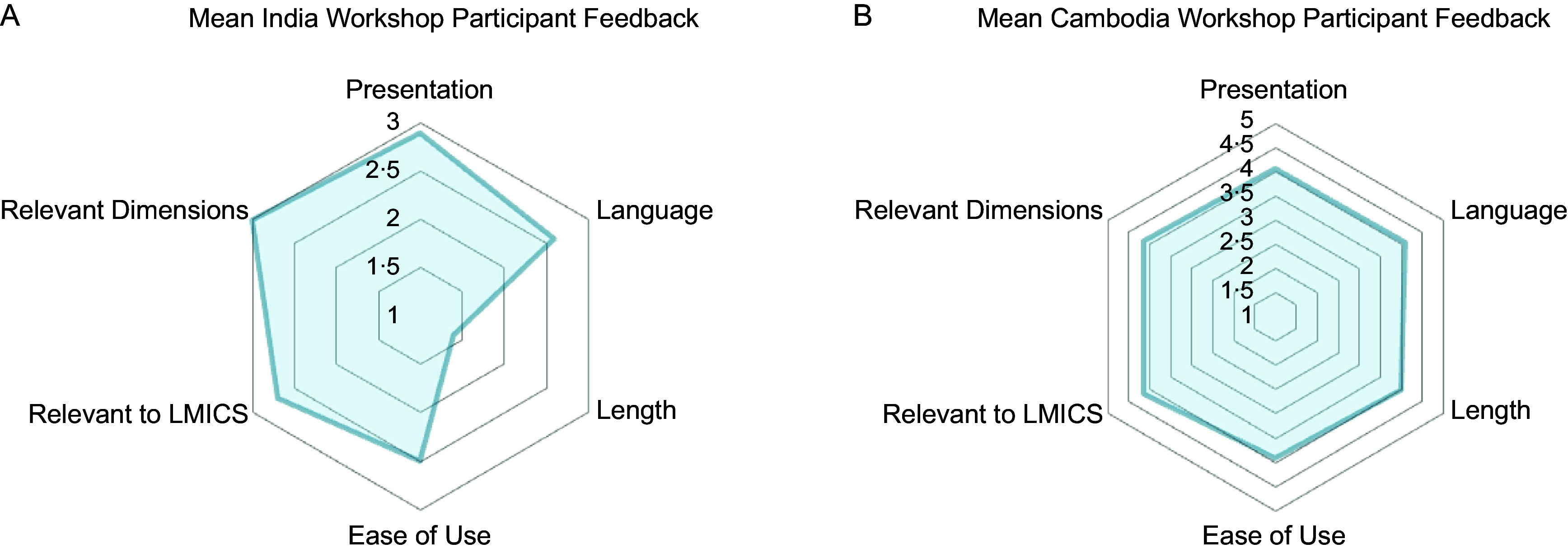
India^1^ and Cambodia^2^ workshop participants’ feedback on different attributes of the Food Environment Perceptions Survey. LMIC, low-and middle-income countries. ^1^The scores from the workshop in India were rated from 1 (viewed negatively) to 3 (viewed positively). ^2^The scores for tool length in India ranged from 1 (very lengthy/somewhat lengthy) to 3 (short). The lengthy and somewhat lengthy response options were collapsed to enable their display on a 3-point scale. ^3^The scores from the workshop in Cambodia were rated from 1 (viewed negatively) to 5 (viewed positively).

During both the workshops in India and in Cambodia, we received extensive feedback from experts on how to further strengthen the survey. In India, over half of the workshop participants rated the overall presentation (*n* 9, 90 %), language (*n* 6, 60 %), ease of use (*n* 6, 60 %), relevancy to LMIC (*n* 7, 70 %) and relevant dimensions (*n* 10, 100 %) positively (i.e. 3 out of 3). However, the respondents felt the tool was somewhat lengthy or lengthy (*n* 6, 60 %) (see Figure 2, Panel A). In Cambodia, which elicited feedback on a more finalised version of the survey, workshop participants rated the survey positively (i.e. strongly agreed or agreed with statements) across all attributes (Figure 2, Panel B). The overwhelming majority gave favourable ratings for the presentation (*n* 13, 100 %), language (*n* 12, 92 %), ease of use (*n* 12, 92 %), relevancy to LMIC (*n* 13, 100 %), length (*n* 12, 92 %) and relevant dimensions (*n* 13, 100 %).

Table 3 summarises the workshop participants’ ratings of each of the sections of the survey instrument. In India, 20 % of participants felt it would be difficult for survey respondents to recollect information about the food labels they see and/or read when purchasing food. In Cambodia, the only question that had fewer than 90 % of participants responding that they agreed or strongly agreed was related to the sufficiency of the information captured on labelling and promotion. We made additional changes to the survey questions, such as refining the wording to improve comprehension and readability, splitting questions into two parts to elicit more specific information, adding/removing response options, etc., after the workshops and prior to piloting the tools.

**Table 3.** Summary of workshop participants’ ratings of each survey section in India and Cambodia

	India workshop scores^*^ (*n* 11)		Cambodia workshop scores^†^ (*n* 13)	
Section	Mean	sd	Mean	sd
Section 1. Accessibility and Availability	2·80	0·40	4·08	0·47
Section 2. Affordability	3·00	0·00	4·15	0·53
Section 3. Convenience	2·82	0·39	4·00	0·39
Section 4. Quality and Safety	2·90	0·30	4·08	0·27
Section 5. Promotion and Labelling	2·73	0·45	4·00	0·55
Section 6. Sustainability	2·82	0·39	4·15	0·36
Section 7. Food Sovereignty	3·00	0·00	*Section removed*	*Section removed*

* The scores from the workshop in India were rated from 1 (viewed negatively) to 3 (viewed positively).

† The scores from the workshop in Cambodia were rated from 1 (viewed negatively) to 5 (viewed positively).

There was extensive feedback about survey food groupings at each stage of survey development. The development and refinement of the food groups underwent numerous iterations. There were differing points of view that made it difficult to reach a consensus. For example, workshop participants in India suggested sub-categories of foods and breaking out starchy staples into their own groups (*n* 3, 23 %). One participant suggested breaking out dark green leafy vegetables into their own food group. During group discussions, participants provided similar feedback and suggested the use of previously established food group classifications (see online supplementary material, Supplemental Table 2 for a description of the final food groups included in the survey). The food groups presented in the workshop in Cambodia were more finalised, and workshop participants provided minimal input on their refinement.

### Pilot testing of the Food Environment Perceptions Survey

Overall, the FEPS survey that was piloted took 50–70 min in rural and peri-urban India and 30–40 min in urban India, whereas in Cambodia, it took an average of 35 min in rural, 32 min in peri-urban and 35 min in the urban settings. In India, we found that respondents were still experiencing challenges responding to the food sovereignty section of the survey; for that reason, we removed the section from the survey shortly after beginning piloting in India. While piloting the tool in India, we also found that the questions related to the food environment dimension of promotion did not seem to be applicable to the rural population as many participants in rural settings did not report being exposed to any food or beverage promotions. However, the section was relevant for all the other populations we piloted and tested the survey with, including rural Cambodia. For that reason, these questions were retained.

Enumerators encountered challenges when implementing the survey in both countries. Questions related to convenience were more difficult for rural respondents to conceptualise, much like the experience with the food promotions module described above. Moreover, questions related to the aspirational aspects of food affordability (e.g. ‘which foods do you wish were cheaper?’) were sometimes difficult for respondents to comprehend.

While conducting preliminary analyses of the pilot data in rural India, we identified issues with the use of the option ‘not applicable’ in several of the questions. This created challenges for data interpretation and analysis. We therefore made changes to the response options included in the programming of the online data collection form. We also made changes to parameters for responses (e.g. only responding in minutes rather than having the option of minutes or hours) to reduce the amount of data cleaning that would be required while also improving the interpretability.

### Description of the finalised version of the Food Environment Perceptions Survey

The final version of the FEPS can be found on the Toolbox website (https://sites.rutgers.edu/food-environment-toolbox/food-environment-perceptions-survey-feps/). The survey consists of 109 questions covering six sections: accessibility and availability (forty-eight questions); affordability (five questions), convenience (seventeen questions); quality and safety (three questions); information, promotion and labelling (sixteen questions); and an optional sustainability section (twenty questions). We included twenty-seven food groups in the final survey instrument; however, four food groups are considered optional.

Based on our piloting data, the survey captured the diversity of food environments that participants were accessing and how those differed across transects. In both India and Cambodia, the number of food environment types accessed was significantly higher in rural (mean 2 (sd 0·85) in Cambodia; mean 3·95 (sd 0·92) in India) compared with peri-urban (mean 1·7 (sd 0·81) in Cambodia; mean 2·6 (sd 0·97) in India) and urban (mean 1·2 (sd 0·43) in Cambodia; mean 2·08 (sd 0·76) in India) settings (*P*-value < 0·001 in India; *P*-value = 0·02 in Cambodia). Moreover, in India, urban (mean 5·2 (sd 2·4)) and peri-urban (mean 5·8 (sd 3·0)) populations accessed more types of food outlets/vendors in the built environment compared with rural participants (mean 2·7 (sd 1·3)) (*P*-value < 0·001). We observed a similar gradient in Cambodia; however, it was not statistically significant (*P* = 0·360). Of note, all (100 %) of the urban participants in India reported accessing food from the online food environment compared with 0 % of the rural participants and 40 % of the peri-urban participants (*P* < 0·001). In Cambodia, only three pilot participants (5 %), from peri-urban/urban settings, reported accessing food from online sources. In India, there were no differences in accessing mobile vendors across the rural, peri-urban and urban transects (*P* = 0·130). However, in Cambodia, all rural participants (100 %) and none (0 %) of the urban participants accessed food from mobile vendors (*P* < 0·001).

In addition to capturing the diversity of food environments that participants were accessing, the survey captured participants’ perceptions of food availability and affordability, including information about which of the food groups they considered to be expensive, which they wished were cheaper and which fluctuated seasonally. The survey also captured participants’ perceptions related to convenience, including which food groups they consider convenient and in which ways (e.g. ‘because it’s easy to prepare’); their exposure to food and beverage-related information, labelling and promotion, including which food groups they were for; and their perceptions related to sustainability and the food groups that they perceive have been impacted by climate variability over the past 10 years. Online supplementary material, Supplemental Table 3 provides an overview of narrative takeaways from our pilot about different dimensions of the food environment by location. Given that we were still making iterative changes to the survey during the piloting in India, we report more data from Cambodia.

## Discussion

This paper describes the co-design, refinement and finalisation of a survey instrument to capture perceptions of food environments among populations living in LMIC, specifically in rural and urban contexts in India and Cambodia. This iterative process of developing, refining and finalising the survey took place over the course of two and a half years in two countries and involved multiple iterative steps and input from research team members, food environment experts and individuals interacting with food environments in the study areas. Given the diversity of food environments accessed in both India and Cambodia, we anticipate that the experiences implementing the survey across transects in these diverse contexts will be relevant to other LMIC settings, with minimal adaptations.

Overall, the FEPS was rated favourably by workshop participants in India and Cambodia. Moreover, our findings suggest that the survey was strengthened over the course of the process of refining and finalising, with each iteration leading to reductions in the amount of time it took to complete, improvements in the flow and improved wording of specific questions. We made numerous changes to the survey throughout the process including both minor changes to wording and terms used and major changes such as removing entire sections of the survey or reconfiguring the way the questions in a specific section were asked.

FEPS was designed with the aim of capturing a wide array of food environments accessed by participants in rural, peri-urban and urban populations in LMIC, given that the food environments that populations access across these gradients of urbanicity can differ substantially^(39–41)^. The finalised survey provided helpful insight into the different food environments that people accessed and how those differed across contexts. Moreover, it provided insight into how the people’s perceptions of different food environment dimensions (e.g. affordability, safety, convenience, etc.) were related to specific food groups, which can inform interventions. As communities are exposed to different food environments and as populations migrate from rural to urban settings, these dimensions may play a more prominent role in their food choices. For example, we found that convenience was difficult for rural populations in India to conceptualise. However, in urban settings, it was relatively easy for respondents to articulate how convenience was influencing their food choices. This likely reflects different time constraints and responsibility within and outside the household for these different population groups, given the time use changes that typically occur when people migrate from rural to more urban settings^(42)^. It might also reflect a wider array of foods to choose from, including more ready-to-eat and snack foods in these settings^(43)^.

We faced many challenges during the process of developing the survey. First, we needed to strike the right balance of covering a sufficient breadth of key dimensions of the food environment, while being time sensitive to respondent burden. Second, we had to refine the most appropriate food groups to include in the survey. Finally, we had to ensure that the questions and their corresponding response options were applicable across settings. One way to overcome the potential time burden challenge for effectively using the instrument in different research is to select the specific sections of the survey that are relevant to the research question being examined, rather than conducting the survey in its entirety. Alternatively, selecting only specific food groups of interest could also help to reduce the amount of time that the survey takes to complete. Lastly, a bigger sample size is needed to conduct more comprehensive analyses; however, this was beyond the scope of this study.

### Conclusion

The finalised version of the survey provides researchers and practitioners with a newly developed survey that provides insight into how consumers’ perceptions of their food environments influence their food choices in LMIC. While it has solely been piloted in two countries to date, we anticipate that it will be relevant to other settings with minimal adaptations. Future piloting in different contexts can continue to refine the survey based on field experiences. Moreover, future work is needed to create corresponding indicators for the survey and to assess the validity of FEPS and its relationship with dietary intakes. Ultimately, it is expected that the knowledge gained from the application of FEPS in diverse contexts will be beneficial to develop solutions to improve food environments for delivering healthy and sustainable diets for all.

## Supporting information

Downs et al. supplementary material Downs et al. supplementary material

## Supplementary material

For supplementary material accompanying this paper, visit https://doi.org/10.1017/S1368980026102043.
