# Supplementary material for: The Food Environment Perceptions Survey: development and piloting of a survey instrument in India and Cambodia to assess consumers’ interactions with diverse food environments
Source: Public Health Nutr. 2026 Feb 13;29(1):e65. doi: 10.1017/S1368980026102043 (PMC13087979; doi:10.1017/S1368980026102043)
Supplement: Downs et al. supplementary material [file S1368980026102043sup001.docx]

**Supplemental Table 1.** Manuscripts, reports, and unpublished survey instruments that were reviewed to inform the development of the Food Environment Perceptions Survey

| Title | Full citation |
| --- | --- |
| Food purchase patterns indicative of household food access insecurity, children’s dietary diversity and intake, and nutritional status using a newly developed and validated tool in the Peruvian Amazon | Ambikapathi, R., Rothstein, J.D., Yori, P.P., Olortegui, M.P., Lee, G., Kosek, M.N. and Caulfield, L.E., 2018. Food purchase patterns indicative of household food access insecurity, children’s dietary diversity and intake, and nutritional status using a newly developed and validated tool in the Peruvian Amazon. Food security, 10(4), pp.999-1011. |
| Informal food environment is associated with household vegetable purchase patterns and dietary intake in the DECIDE study: Empirical evidence from food vendor mapping in peri-urban Dar es Salaam, Tanzania | Ambikapathi, R., Shively, G., Leyna, G., Mosha, D., Mangara, A., Patil, C.L., Boncyk, M., Froese, S.L., Verissimo, C.K., Kazonda, P. and Mwanyika-Sando, M., 2021. Informal food environment is associated with household vegetable purchase patterns and dietary intake in the DECIDE study: Empirical evidence from food vendor mapping in peri-urban Dar es Salaam, Tanzania. Global food security, 28, p.100474. |
| Perceptions of a healthier neighborhood food environment linked to greater fruit and vegetable purchases at small and non-traditional food stores | Barnes TL, Lenk K, Caspi CE, Erickson DJ, Laska MN. Perceptions of a healthier neighborhood food environment linked to greater fruit and vegetable purchases at small and non-traditional food stores. J Hunger Environ Nutr. 2019;14(6):741-761. doi: 10.1080/19320248.2018.1549518. Epub 2018 Nov 23. PMID: 31798762; PMCID: PMC6886741. |
| Perceptions of the food shopping environment are associated with greater consumption of fruits and vegetables | Blitstein, J.L., Snider, J. and Evans, W.D., 2012. Perceptions of the food shopping environment are associated with greater consumption of fruits and vegetables. Public health nutrition, 15(6), pp.1124-1129. |
| Food Sovereignty Indicators for Indigenous Community Capacity Building and Health | Blue Bird Jernigan, V., Maudrie, T.L., Nikolaus, C.J., Benally, T., Johnson, S., Teague, T., Mayes, M., Jacob, T. and Taniguchi, T., 2021. Food sovereignty indicators for Indigenous community capacity building and health. Frontiers in Sustainable Food Systems, p.307. |
| Improving Nutrition in Nepal: An analysis of food environments, behavioral determinants of consumption, and costs of a multisectoral nutrition program | Choo, E. (2023). Improving Nutrition in Nepal: An analysis of food environments, behavioral determinants of consumption, and costs of a multisectoral nutrition program University of Washington. |
| Leverage Points To Increase The Availability And Affordability Of Nutrient-Dense Foods: A 'Food Environment Experience' Survey In East Lombok (Indonesia) And Hyderabad (India) | Cooper, G. S., Zahra, N. L., Konapur, A., Davies-Kershaw, H., Dominguez-Salas, P., Fahmida, U., Ferguson, E., Frongillo, E., Häsler, B., Pramesthi, I. L., Rowland, D., Selvaraj, K., Munikumar, M., Banjara, S., Shankar, B., Sudibya, A., & Kadiyala, S. (2023). *Leverage Points To Increase The Availability And Affordability Of Nutrient-Dense Foods: A 'Food Environment Experience' Survey In East Lombok (Indonesia) And Hyderabad (India),* ANH2023. |
| Food Choice Questionnaire (FCQ) revisited | Fotopoulos, C., Krystallis, A., Vassallo, M. and Pagiaslis, A., 2009. Food Choice Questionnaire (FCQ) revisited. Suggestions for the development of an enhanced general food motivation model. Appetite, 52(1), pp.199-208. |
| Access to Healthful Foods among an Urban Food Insecure Population: Perceptions versus Reality | Freedman, D.A. and Bell, B.A., 2009. Access to healthful foods among an urban food insecure population: perceptions versus reality. Journal of Urban Health, 86(6), pp.825-838. |
| Development of the perceived nutrition environment measures survey | Green, S. H., & Glanz, K. (2015). Development of the perceived nutrition environment measures survey. *American journal of preventive medicine*, *49*(1), 50-61. |
| Food Enviornment Analysis | Grude, A. (2020). *Food Environment Analysis: India Report*. ACDI/VOCA. |
| The 5 A's Approach for Contextual Assessment of Food Environment | Konapur, A., Gavaravarapu, S.M. and Nair, K.M., 2022. The 5 A's Approach for Contextual Assessment of Food Environment. Journal of Nutrition Education and Behavior. |
| Test–retest reliability of a questionnaire measuring perceptions of neighborhood food environment | Ma, X., Barnes, T.L., Freedman, D.A., Bell, B.A., Colabianchi, N. and Liese, A.D., 2013. Test–retest reliability of a questionnaire measuring perceptions of neighborhood food environment. Health & place, 21, pp.65-69. |
| Between the city and the farm: food environments in artisanal mining communities in Upper Guinea | Nordhagen, S., Fofana, M.L., Diallo, S., Songbono, J.L., Stokes-Walters, R., Zhang, L.X., Klemm, R. and Winch, P.J., 2022. Between the city and the farm: food environments in artisanal mining communities in Upper Guinea. Public health nutrition, 25(2), pp.368-380. |
| Validity of a questionnaire measuring motives for choosing foods including sustainable concerns | Sautron, V., Péneau, S., Camilleri, G.M., Muller, L., Ruffieux, B., Hercberg, S. and Méjean, C., 2015. Validity of a questionnaire measuring motives for choosing foods including sustainable concerns. Appetite, 87, pp.90-97. |
| Consumers’ knowledge and safety perceptions of food additives: Evaluation on the effectiveness of transmitting information on preservatives | Shim, S.M., Seo, S.H., Lee, Y., Moon, G.I., Kim, M.S. and Park, J.H., 2011. Consumers’ knowledge and safety perceptions of food additives: Evaluation on the effectiveness of transmitting information on preservatives. Food Control, 22(7), pp.1054-1060. |
| An Emergent Framework of the Market Food Environment in Low-and Middle-Income Countries | Toure, D., Herforth, A., Pelto, G.H., Neufeld, L.M. and Mbuya, M.N., 2021. An Emergent Framework of the Market Food Environment in Low-and Middle-Income Countries. Current developments in nutrition, 5(4), p.nzab023. |
| Is the objective food environment associated with perceptions of the food environment? | Williams, L.K., Thornton, L., Ball, K. and Crawford, D., 2012. Is the objective food environment associated with perceptions of the food environment?. Public health nutrition, 15(2), pp.291-298. |
| Food safety and food quality perceptions of farmers’ market consumers in the United States | Yu, H., Gibson, K.E., Wright, K.G., Neal, J.A. and Sirsat, S.A., 2017. Food safety and food quality perceptions of farmers’ market consumers in the United States. Food Control, 79, pp.266-271. |
| Searching for a measure integrating sustainable and healthy eating behaviors. | Żakowska-Biemans, S., Pieniak, Z., Kostyra, E. and Gutkowska, K., 2019. Searching for a measure integrating sustainable and healthy eating behaviors. Nutrients, 11(1), p.95. |

**Supplemental Table 2**. Finalized food groups used in the Food Environment Perceptions Survey

| **Food Groups** | **Examples** |
| --- | --- |
| **Core food groups** | |
| Starchy staples | **Examples:** Barley, bread, breadfruit, brown rice, bulgur, cassava meal, cassava/yucca, corn/maize (dried), flours of the whole grains, fonio, fortified flours, green banana maize flour/meal, millet, noodles, oats, pasta, plantains, popcorn, potato, puffed rice, purple sweet potato, quinoa, rice flakes, rice flour, sorghum, taro, turnip, white rice, white sweet potato, whole grain bread |
| Legumes/pulses | **Examples:** Black eye pea/cowpea, chickpea/garbanzo bean, common black bean, dried fava beans, dried peas, lentils, mung beans, pigeon pea, soybeans, soymilk, tempeh, tofu, white beans |
| Nuts and seeds | **Examples:** Almonds, cashews, chestnuts, flax seeds, flavored nuts and seeds, hazelnuts, macadamia nuts, peanut butter, peanuts, pecans, pine nuts, pistachio, pumpkin seeds, nut and seed butters, sesame seeds, sunflower seeds, tahini, walnut, watermelon seeds |
| Milk, yogurt, and fresh cheese | **Examples:** Camel milk, cottage cheese, curd, evaporated milk (unsweetened condensed milk), fermented milk, fresh whole milk from buffalo, fresh whole milk from cow, fresh whole milk from goat, kefir, local cheese, milk powder, mozzarella cheese, skim or low-fat milk, UHT milk (unflavored and unsweetened), yogurt |
| Eggs | **Examples:** Chicken egg, duck egg, quail egg |
| Red meat | **Examples:** Beef, beef liver or other organ meat, buffalo, goat, lamb, pork, sheep (mutton), veal, venison |
| Poultry and game meat | **Examples:** Camel, chicken, chicken liver or other organ meat, duck, frog, goose, guinea fowl, pigeon, quail, rabbit, rat, turkey, turtles |
| Fish and seafood | **Examples:** Canned fish, canned sardines, crab, dried fish, dried seafood, eel, fermented fish, fish from sea, freshwater fish, shrimp, small fish, smoked fish, squid, tuna |
| Processed meat | **Definition:** The World Health Organization defines processed meat as “…meat that has been transformed through salting, curing, fermentation, smoking, or other processes to enhance flavour or improve preservation. Most processed meats contain pork or beef, but processed meats may also contain other red meats, poultry, offal, or meat by-products such as blood.”  **Examples:** Bacon, bologna, canned meat, chorizo sausage, cold cuts/luncheon meat, corned beef, dried meat, ham, hot dog, pâté, pepperoni, salami, sausage |
| Vitamin A-rich vegetables | **Examples:** Butternut squash, carrots, dried orange-fleshed vegetables, fermented orange-fleshed vegetables, frozen orange-fleshed vegetables, orange-fleshed squash, pumpkin, red/yellow pepper (sweet), sweet potatoes (orange inside) |
| Dark green leafy vegetables | **Examples:** Amaranth (green), arugula, baobab leaves, bean leaves, bok choy, broccoli, cassava greens, chard, Chinese cabbage, collard greens, cowpea leaves, dried dark leafy greens, eggplant leaves, fermented dark leafy greens, frozen dark leafy greens, grape leaves, hibiscus leaves, jute mallow, kale, lettuce (medium and dark green), moringa greens, mustard greens, pumpkin leaves, sorrel, spinach, sweet potato leaves, taro leaves, water spinach, watercress, wild leaves |
| Other vegetables | **Examples:** African eggplant, beet, bitter gourd, bottle gourd, cabbage, cauliflower, celery, chayote, corn (fresh), cucumber, dried other vegetables, eggplant, fermented other vegetables, frozen other vegetables, green beans, green pepper (capsicum), ivy gourd, lettuce (light green), long beans, mushrooms, okra, radish, tomatoes, zucchini |
| Vitamin A-rich fruits | **Examples:** Apricot, cantaloupe (ripe), dried orange-flesh fruits, fermented orange-fleshed fruit, frozen orange-fleshed fruit, mamey sapote, mango (ripe), papaya (ripe), passion fruit, persimmon |
| Other fruits | **Examples:** Apple, avocado, banana, baobab fruit, berries, cherries, coconut flesh, custard apple (sweetsop), dates, dragon fruit, dried other fruit, fermented other fruit, figs, frozen other fruit, grape fruit, grapes, green mango, green papaya, guava, jackfruit, jujube, lychee, mandarin, mulberries, nectarine, orange, peach, pear, pineapple, plum, pomegranate, pomelo, prickly pear, rambutan, sapota/sapodilla, soursop, star fruit, strawberry, watermelon, wild fruits |
| Fats and oils | **Classification:** Animal fats/oils  **Examples:** butter, ghee, lard/pork fat |
|  | **Classification:** Plant fats/oils  **Examples:** coconut oil, groundnut/peanut oil, other plant-based oils, palm oil, safflower oil, soybean oil, sunflower oil, vegetable oil |
|  | **Classification:** Hydrogenated fats, flavored spreads, etc.  **Examples:** Flavored butter, fortified margarine/vegetable fat, margarine |
| Sweets | **Classification:** Grain-based  **Examples:** baked churros, baked doughnuts, cake, cookies/biscuits, fried dough in syrup, pastries, sweet bread, sweet crepe, wafers |
|  | **Classification:** Other sweets  **Examples:** Candy, chocolate, pudding, rice pudding |
|  | **Classification:** Ice cream  **Examples:** frozen yogurt, gelato, ice cream, ice pops, popsicles, sorbet |
| Sweet drinks | **Classification:** Sweet tea/coffee/cocoa/milk-based drinks  **Examples:** 3-in-1 instant coffee, bubble tea, chocolate drinks, chocolate frappe, coffee frappe, flavoured kefir, flavoured milk drinks, fortified milk drinks, milo/Nesquik, sweetened coffee drinks, sweetened condensed milk, sweetened tea |
|  | **Classification:** Fruit juice, smoothies, and other fruit-based sweet drinks  **Examples:** bissap/hibiscus drink, fresh fruit juices, fresh lemonade, fresh sugarcane juice, fruit flavoured drinks, fruit smoothies, ginger drink |
|  | **Classification:** Sodas, energy drinks, sports drinks, etc.  **Examples:** Carbonated lemonade, carbonated malt drinks, diet soft drinks, energy drinks, soft drinks, sports drinks (drinks with electrolytes) |
| Ultra-processed salty snacks | **Examples:** Chips, potato chips, puffs |
| Ultra-processed ready-to-eat/heat foods | **Examples:** Frozen chicken nuggets and sticks, frozen chips/fritters/wedges, frozen dumplings, frozen meals, frozen meat balls, frozen mozzarella sticks, frozen pizza, frozen sausages, indomie noodles, instant noodles, instant soups, Maggi noodles, packaged breads, packaged sugar-coated cereals, packaged sweet bread |
| Prepared foods by street vendors and restaurants | **Classification:** Deep-fried foods  **Examples:** bean fritters, French fries, fried bananas, fried cassava, fried chicken, fried dough, fried fish, fried plantain, fried pork rinds, fried samosa, fried sweet potato, fritters |
|  | **Classification:** Mixed-dishes, soups, and meals  **Examples:** meat and rice, prepared mixed-dishes served by street vendors |
|  | **Classification:** Prepared salads and other fruit- or vegetable-based dishes  **Examples:** Locally prepared salads |
| Salt, MSG, and salty sauces | **Examples:** Bouillon cubes or powder (i.e., vegetable stock cubes, chicken stock cubes), iodized salt, MSG, other salty sauces and seasonings |
| Simple sugars | **Examples:** Brown sugars, coconut sugar, honey, sugar, syrup |
| Fast food | Foods bought from chain restaurants (i.e., Burger King, Dominos, KFC, McDonald’s, Pizza Hut, Subway, Taco Bell, etc.) such as:  **Examples:** burgers, French fries, fried chicken, pizza |
| **Optional food groups** | |
| Bottled water | **Examples:** Tetra-packed water |
| Alcohol | **Examples:** Beer, homebrewed alcohol, liquor, palm wine |
| Fortified mixes | **Examples:** Fortified breakfast mixes, infant mixes |
| Spices, tea, coffee, and condiments | **Examples:** Coconut milk, dried or fresh chilies, fresh or dried spices and herbs, garlic, ginger root, instant coffee powder, roasted cocoa beans/powder, roasted coffee beans/powder, sweetened condensed milk, tea leaves |
| Insects | Ants, camel, crickets, flying termites, grasshoppers, locusts, snails, spiders |
| **References** | |
| FAO and FHI 360. 2016. Minimum Dietary Diversity for Women: A Guide for Measurement. Rome: FAO. | |
| WHO International Agency for Research on Cancer (IARC). 2018. Red meat and processed meat. Lyon, France. | |

**Supplemental Table 3**. An overview of key findings from the pilot data collection in India and Cambodia

| **Food environment dimension** | **Key findings from India and Cambodia pilot data*** |
| --- | --- |
| Accessibility and availability | *Key findings from India*: Most (85%; n=11) of urban participants strongly agreed with the statement about their satisfaction with the availability and/or variety of food in their community as compared to 30% (n=3) of peri-urban and 48% (n=10) of rural participants (p=0.065)  *Key findings from Cambodia*: There was a statistical difference in participant satisfaction with the availability and/or variety of foods available within the community across settings: 66% of participants in both rural (n=10) and peri-urban (n=10) settings only somewhat agreed with the statement related to satisfaction about availability whereas 80% of high-income urban (n=12) and 87% of low-income urban (n=13) strongly agreed (p<.001). |
| Affordability | *Key findings from India*: While a third of rural participants (n=7) reported starchy staples to be relatively expensive, none of the urban or peri-urban participants did (p=0.0.01). A higher proportion of rural (76%; n=16) and peri-urban (40%; n=4) participants found ‘other vegetables’ to be relatively expensive (p=0.002). Given changes to the survey during the piloting, we only have data from a small number (n=13) of urban participants. Of those participants, five indicated they would purchase more fruits and/or vegetables and one reported they would purchase more processed and freshly prepared foods and/or beverages with higher income. Fruits and vegetables were the foods that participants indicated fluctuated in price across seasons in rural, urban, and peri-urban settings, with no statistical differences across settings.  *Key findings from Cambodia*: Unprocessed meat from ruminants (ranging from 67% in high-income urban (n=10) to 93% in rural (n=13)/peri-urban (n=14)) and non-ruminants (ranging from 73% in high-income urban (n=11) to 87% in rural (n=13)/peri-urban (n=13)) were perceived as relatively expensive across all settings. For all other food groups, only a relatively small percentage of participants (<20%) reported the food group as being relatively expensive, with the exception of peri-urban (40%; n=6) and low-income urban (27%; n=4) participants reporting that starchy staples were relatively expensive. There were significant differences in the proportion of participants from different settings that would purchase more red meat from both ruminants (p=0.013) and meat from non-ruminants, fish, and/or poultry (p=0.027) if their income was higher, with the lowest proportions among the urban high income (unprocessed red meat (ruminant): 46.7% n=7; unprocessed meat (non-ruminant), fish, and poultry: 53.3% n=8) as compared to rural participants (n=4; 93% for both). The only other food group where over 20% of participants indicated they would purchase more with higher income was milk and milk products among low-income urban (n=3; 30%) and other fruit among high-income urban (n=4; 26.7%). When asked how they would change their food purchasing if their income was higher, buying more variety was cited by the majority of low-income urban (n=9; 60%), peri-urban (n=11; 73%) and rural participants (n=9; 60%), whereas an equal proportion (n= 6; 40%) of high-income urban participants said they would buy more variety as would buy higher quality foods (n=6; 40%). The food groups for which participants reported the most seasonal variation were unprocessed red meat (ruminants) (ranging between 53% (n=8) among rural and urban high-income to 67% (n=10) among peri-urban participants) and all types of fruits and vegetables; with rural participants reporting less seasonal variation in the affordability of fruits than other settings (p=0.005). Participants reported purchasing eggs, dark leafy greens, and vegetables due to their low cost among all settings and edible oils among low-income urban. |
| Convenience | *Key findings from India*: Convenience questions were difficult for the rural population to conceputalize. We therefore made significant changes to this section prior to piloting with peri-urban and urban populations. For that reason, we only report data for most questions from these settings. Convenience was primarily thought of in terms of ease of access or availability (71.4% of peri-urban (n=5); 84.6% of urban (n=11)), followed by ease of clean-up among peri-urban respondents (57.1%; n=4), ease of preparation (42.9% (n=3) of peri-urban and 61.5% (n=8) of urban), and vendors being available at convenient hours (46.2% of peri-urban and urban respondents). Half of urban participants (n=4) indicated that starchy staples and legumes, pulses, nuts, and seeds were convenient due to their ease of preparation. All of rural participants reported that a female member of the household prepared food most of the time, while 85% (n=11) of urban households did with the remaining participants indicating that hired personnel prepared most of the food. Between 20-30% of participants stated that male members of the household decided what to purchase, with the remaining being female members. A higher proportion of urban participants (46%; n=6) than rural (29%; n=6) and peri-urban (30%; n=3) participants reported sometimes feeling constrained for meal preparation. Overall, participants spent an average of just over two hours per day (ranging from 1-4 hours per day) preparing food for the household  *Key findings from Cambodia*: We found significant differences across settings in terms of viewing convenience as ease of preparation (33% (n=5) high-income urban; 93% (n=14) low-income urban; 100% (n=15) peri-urban; and 87% (n=13) rural; p<.001). High-income urban (40%; n=6) and peri-urban (33%; n=5) participants also reported pre-prepared, packaged, or low perishability foods as being convenient more than low-income urban and rural (6.7%; n=1) (p=0.043). While convenience was conceptualized in terms of accessibility and availability among all settings, there were some significant differences in the foods that were viewed as easily accessible. For example, a higher proportion of participants living in rural settings found vegetables accessible than in other settings (p=0.023), a higher proportion of peri-urban participants indicated eggs were convenient due to their accessibility (p=0.027), and processed and freshly prepared foods and beverages (p=0.016) as well as ultra-processed were reported as being convenient due to accessibility by a higher proportion of high-income urban participants (p=0.016). Foods that were considered easy to prepare included eggs, dark leafy greens by participants in all settings, unprocessed red meat (ruminants), particularly among high-income urban participants (p=0.003), unprocessed meat (non-ruminant), fish and/or poultry (p=.023) and other vegetables, particularly among low- and high-income urban participants, and ultra-processed salty foods and/or beverages mostly among peri-urban populations (p=0.010). For the most part, female members of households were responsible for preparing food across all settings in Cambodia. Interestingly, three high-income urban participants (20%) reported not preparing any food in the home (e.g., purchasing it from outside the home) over the previous 30 days. While women were most likely to decide which foods to prepare, a couple men in low-income urban and peri-urban households were decision makers (p=0.048). There was a significant difference in the amount of time spent preparing food across settings as well, with rural households spending significantly more time on average (115.3 min +/- 69.8) as compared to high income urban participants (56.9 min +/- 51.2) (p=.035). There was a significant difference in how often participants felt time constraints across settings (p=.012), as well as the strategies used to overcome these time constraints. Urban (low- (92%; n=11) and high-income (62%; n=8)) and peri-urban participants (60%; n=3) often consumed convenience foods (e.g., ready to eat, packaged, etc.) to overcome these constraints as compared to rural participants (17%; n=2) (p=.003). Urban participants (low- (50%; n=6) and high-income (31%; n=4)) also reported preparing food in bulk and storing it for future use more often than peri-urban (0%) and rural participants (0%) (p=.017). Meanwhile, rural (67%; n=8) most often reported doing nothing to overcome the time constraints compared to only one or two participants reporting this from other settings (p=.003). |
| Food safety | *Key findings from India*: We made several changes to the food safety and hygiene section during the piloting, making it only possible to examine the data from the urban respondents in India, where we found that vegetables were the main concern related to cleanliness followed by prepared foods from street vendors.  *Key findings from Cambodia*: In Cambodia, where we were able to analyze data across settings, we found unprocessed meat (non-ruminant), fish and/or poultry, dark leafy greens, and other vegetables to be the food groups that participants were concerned about regarding the cleanliness. We also found that other fruit (p=0.013) and vitamin A rich fruits and vegetables (p=0.003) were of concern in the urban but not rural and peri-urban settings. Participants across all settings indicated that they were concerned about pesticides and chemicals for most food groups; exceptions included milk and milk products, edible oils/fats, simple sugars, condiments, and fast food. In contrast, few participants reported being concerned about adulteration, preservatives/additives, or contamination, with the exception of ultra-processed sweet foods and/or beverages where between 53-73% of participants across settings reported being concerned and ultra-processed salty snacks where 60% of participants in each setting were concerned. |
| Information | *Key findings from India*: An earlier version of the survey did not make the distinction between exposure to information about food and promotions. For that reason, we do not have data related to exposure to different sources of information for India.  *Key findings from Cambodia*: In the updated version of the survey in Cambodia, we asked about exposure to information related to health, safety, and the quality of food. We found a difference in exposure across settings with 93% of high-income urban participants (n=14) being exposed to information as compared to 87% (n=13) in low-income urban, 67% (n=10) in peri-urban, and 40% (n=6) in rural settings (p=.005). Across all settings most participants received the information from social media (ranging from 60% of participants in peri-urban settings to 93% in high-income urban settings). The information was mostly for animal-source foods, fruits, vegetables, and ultra-processed foods across all settings. |
| Promotion | *Key findings from India*: A higher proportion of urban (85%; n=11) compared to peri-urban (30%; n=3) and rural participants (48%; n=10) reported being exposed to food and/or beverage promotions (p<.001). There were differences in exposure to promotions across settings with peri-urban and urban populations being exposed to significantly more social media (80% (n=8) vs 19% (n=4); p=.004), TV (14% (n=3) rural, 60% (n=6) peri-urban, 54% (n=7) urban; p=.028), newspapers, billboards, posters/pamphlets, than rural areas. Sampling at grocery stores or malls only happened in urban areas; however, free samples sent with newspapers were present across all settings (47.6% (n=10) in rural, 90% (n=9) in peri-urban, 85% (n=11) in urban; p=.019). Promotions through delivery apps were only in peri-urban (40%; n=4) and urban (31%; n=4) settings (p=.011). On the other hand, promotions through door-to-door campaigns (9.5% (n=2); p=.020) or by people singing jingles were only experienced by rural respondents (14.3% (n=3); p=.008), albeit a very small number. Given changes in the survey, we only had data about the content of the promotion that influenced respondents to buy specific foods from urban populations  *Key findings from Cambodia*: In Cambodia, all participants, regardless of setting, indicated that they were exposed to food and/or beverage promotions. We found significant differences in food and/or beverage promotion via social media exposure across settings with 60% of rural (n=9) and peri-urban participants (n=9) being exposed to promotion as compared to 93% (n=14) of both low- and high-income urban participants (p=0.025). Exposure to other mediums of promotions was limited, except for TV promotions among peri-urban (33%; n=5) and rural (40%; n=6) participants. The promotions were mostly for ultra-processed foods and alcohol across all settings and participants indicated that the game/sweepstakes/prize promotions were the promotions that influenced them to purchase the associated products most often. |
| Labeling | *Key findings from India*: Whereas all peri-urban and most urban (92%; n=12) participants reported that the packaged foods they were exposed to had labels, less than a third (29%; n=6) of rural participants did (p<.001). Of those who were exposed to labels, there was a significant difference in respondents reporting that they read the labels on packaged foods across the different settings with only one rural respondent reporting to read labels as compared to 50% of peri-urban (n=5) and 77% or urban respondents (n=10) (p<.001), with the expiry date being the most reported content on the label that they recall observing.  *Key findings from Cambodia*: A similar pattern was observed where fewer rural participants reported reading labels (47%; n=7) as compared to peri-urban (60%; n=60), and urban (low- (87%; n=13) and high-income (93%; n=14)) (p=0.041). Like India, the expiry date was the most reported information that participants read across all settings followed by the country of origin. |
| Sustainability | *Key findings from India*: Most participants reported observing changes in the foods available in shops over the past 10 years, across all settings. These changes included increase availability of food, increased number of varieties in urban (69%; n=9) and peri-urban (40%; n=4) but not rural (0%) settings (p<.001), decreased number of varieties in urban (85%; n=11) and peri-urban (50%; n=5) but not rural (0%) settings (p<.001), and increased amount of local food in urban (46%; n=5) and peri-urban (50%; n=4) as compared to rural (7%; n=1) (p=0.045). Rural participants reported changes in wild game in their community (52%; n=11); however, none of the peri-urban or urban participants did (p<0.001). A similar pattern was observed for fish and/or seafood with only rural participants (29%; n=6) observing changes (p=0.028). A higher proportion of rural participants (76%; n=16), as compared to urban (54%; n=7) and peri-urban (30%; n=3) participants reported observing changes in temperature, precipitation, or overall weather patterns over the past 10 years (p<0.001). We added questions to specify how these changes were impacting specific foods in the subsequent version of the survey that was piloted in Cambodia. Most urban participants (77%; n=10) strongly agreed with the statement related to concern about the amount of plastic packaging as compared to peri-urban (30%; n=3) and rural (19%; n=4); however, it was not significant (p=.107)  *Key findings from Cambodia*: Nearly all participants, across all settings, reported observing changes to the foods available in shops over the past 10 years. These changes included increased availability and variety across most food groups (except simple sugars), increased prices (mostly for fresh foods such as animal-source foods, fruits, and vegetables; and staple foods among urban and peri-urban participants only), increased imported foods, increased local foods, and worse quality foods (unprocessed meat (both ruminant and non-ruminant), fish, and poultry, vegetables, and fruits). We found significant differences across settings in terms of changes in wild meat in communities, with only participants in rural (67%; n=10) and peri-urban (80%; n=12) settings indicating a change (p<0.001), which was primarily a decrease in its availability. Nearly all participants, across all settings, reported changes to fish and seafood within the community; however, there were differences in the ways in which it had changed. A higher proportion of urban (high-income (64%; n=7) and low-income (91%; n=10)) and peri-urban participants (40%; n=6) observed increased availability as compared to the rural participants (8.3%; n=1) (p<.001), while a higher proportion of rural participants indicated that there was decreased availability (p=0.013). Urban participants also indicated having to travel less far to reach fish and/or seafood compared to rural participants (p=0.048). Across all settings, participants indicated a decline in the species variety over the past 10 years; however, there were differences across settings ranging from 36% (n=4) of low-income urban to 87% (n=13) peri-urban participants (p=0.037). In terms of changes in wild plants, only peri-urban (80%) and rural participants (53%) reported changes (p<0.001). Of those who indicated changes in access to wild plants, all peri-urban, and 88% (n=7) of rural, participants indicated their availability had decreased. A few (n=3) of the rural participants indicated that they had to travel further to access wild plants. Three quarters of peri-urban and half of rural participants indicated that there were fewer varieties of wild plants. Most (80%; n=12) of rural participants observed changes in the number of pests and disease of crops, fish, and/or farm animals over the past 10 years whereas only a third of peri-urban participants, and one urban participant did (p<0.001). Starchy staples and vegetables were the most commonly impacted food groups. All but one participant reported observing changes in temperature, precipitation, or overall weather patterns in their community over the past 10 years, all of whom observed an increase in temperatures. Over half of participants from all settings observed less rainfall and delayed rainfall was reported by over half of low-income urban (60%; n=9) and rural (53%; n=8) participants and all of peri-urban participants (p<.007). The food groups that over half of participants across settings reported being impacted by these weather changes included starchy staples, legumes, nuts and seeds (with the exception of rural participants where only 27% reported), dark leafy green vegetables, other vegetables, vitamin A rich yellow and orange fleshed fruits and vegetables (with the exception of rural participants (47%)), and other fruits. Unprocessed red meat (ruminants) was reportedly impacted more by urban (low- (71%; n=10) and high-income (53%; n=8)) as compared to peri-urban (13%; n=2) and rural (40%; n=6) participants (p<.014). Unprocessed meat (non-ruminant), fish and/or poultry was reportedly impacted by a higher proportion of urban (low- (67%; n=10) and high-income (71%; n=10) than peri-urban (13%; n=2) and rural participants (47%; n=7) (p=.006). All participants from urban and peri-urban settings reported that dark leafy greens and other vegetables were impacts, whereas fewer rural participants did (dark leafy greens: 80%; n=12 (p=.026; other vegetables: 87%; n=13; p=.108). Most participants across settings somewhat agreed with the statement “I am concerned about the plastic packaging in the foods that I buy. There were statistical differences across settings with regards to the statement about being worried that the way some foods are produced is harmful to the environment (p=0.025). More specifically, 60% of low-income urban (n=9) and peri-urban participants (n=10) strongly agreed, as compared to 20% of high-income urban (n=3) and 47% (n=7) of rural participants. There were also statistical differences in the statement about having enough information to make environmentally friendly choices when it comes to purchasing and consuming food (p=0.031). More specifically, we found that the highest proportion of rural and low-income urban participants either strongly or somewhat agreed as compared to high-income urban and rural. None of the urban participants reported using kitchen scraps as compared to 33% (n=5) of peri-urabn and 40% (n=6) of rural (p=0.009). |

*Given the iterative nature of making changes to the survey throughout the piloting process, we report more data from Cambodia than India given that a more finalized version of the survey instrument was used across settings there
